# Genome-Wide Analysis of Human Disease Alleles Reveals That Their Locations Are Correlated in Paralogous Proteins

**DOI:** 10.1371/journal.pcbi.1000218

**Published:** 2008-11-07

**Authors:** Mark Yandell, Barry Moore, Fidel Salas, Chris Mungall, Andrew MacBride, Charles White, Martin G. Reese

**Affiliations:** 1 Eccles Institute of Human Genetics, University of Utah and School of Medicine, Salt Lake City, Utah, United States of America; 2 Omicia, Emeryville, California, United States of America; 3 Lawrence Berkeley National Laboratory, Berkeley, California, United States of America; University of California, Los Angeles, United States of America

## Abstract

The millions of mutations and polymorphisms that occur in human populations are potential predictors of disease, of our reactions to drugs, of predisposition to microbial infections, and of age-related conditions such as impaired brain and cardiovascular functions. However, predicting the phenotypic consequences and eventual clinical significance of a sequence variant is not an easy task. Computational approaches have found perturbation of conserved amino acids to be a useful criterion for identifying variants likely to have phenotypic consequences. To our knowledge, however, no study to date has explored the potential of variants that occur at homologous positions within paralogous human proteins as a means of identifying polymorphisms with likely phenotypic consequences. In order to investigate the potential of this approach, we have assembled a unique collection of known disease-causing variants from OMIM and the Human Genome Mutation Database (HGMD) and used them to identify and characterize pairs of sequence variants that occur at homologous positions within paralogous human proteins. Our analyses demonstrate that the locations of variants are correlated in paralogous proteins. Moreover, if one member of a variant-pair is disease-causing, its partner is likely to be disease-causing as well. Thus, information about variant-pairs can be used to identify potentially disease-causing variants, extend existing procedures for polymorphism prioritization, and provide a suite of candidates for further diagnostic and therapeutic purposes.

## Author Summary

There exists a superabundance of human sequence variations. Testing every sequence variant for association with human disease is often infeasible, as studies must be very large—and hence expensive—to overcome the statistical penalties used to control for multiple tests. A common alternative is to assay only a subset of sequence variants for which there are prior reasons to believe they may be disease-causing. Sequence variants that change conserved amino acids, for example, are often disease-causing. As an adjunct to this approach, we have explored the potential of variants that occur at homologous positions within paralogous human proteins as a means of identifying disease-causing DNA sequence variations. We find that DNA sequence variants co-occur at aligned amino acid pairs more frequently than expected by chance, suggesting that similar functional constraints on paralogous proteins result in coordinated distributions of variants along their lengths. Moreover, if one member of a variant-pair is disease-causing, its partner is likely to be disease-causing as well. These facts provide new avenues for the identification of disease-causing sequence variations.

## Introduction

The publicly available dbSNP database [1] contains approximately 12 million unique human sequence variants, few of which are associated with any particular phenotype or disease. Large-scale association studies often produce hundreds of first “hits” in the form of individual SNPs or haplotypes that then need to be “characterized” for a potential role in a disease phenotype; see [2]–[4]. Currently, there exist few methods for prioritizing variations in a locus for use in association studies or to determine post-facto whether an association found in a genome wide association study is likely to hold up to further testing.

The large number of uncharacterized SNPs has driven the development of computational methods aimed at identifying those variations likely to cause disease. To date, the most successful approaches to *in silico* SNP characterization have been protein-based and comparative in nature, namely SIFT [5]–[7] and PolyPhen [8]–[9]. These approaches, often collectively referred to as Amino Acid Substitution, or AAS, approaches (for a review, see [4]), examine non-synonymous changes in human proteins in the context of multiple alignments of homologous proteins from other organisms. The assumption is that variants effecting amino acid replacements rarely seen in a given column of the multiple alignment will adversely impact protein function and perhaps cause disease [4]. However, the complex relationship between sequence conservation, protein function and disease poses a difficult problem for comparative approaches. For some proteins, conservative changes in poorly conserved regions may be the only tolerated changes, and even these may have severe phenotypic consequences; in other cases, even non-conservative changes that destroy protein function may not be disease-causing, especially for non-essential, redundant genes. These considerations suggest that algorithms for the identification of disease-causing variations based upon trends in protein sequence conservation will miss many particular instances.

Given these issues, we wondered whether paralogous human genes might provide a source of additional information for *in silico* variation characterization, one complementary to AAS approaches such as SIFT [5]–[7] and PolyPhen [8],[9]. By ‘paralogous genes’ we mean “Genes that have arisen by gene duplication events in an organism and are transmitted to offspring as a gene family”[10]. By this criterion, over half of human genes have at least one paralog. The advantage of using paralogous genes is that information from them is uncomplicated by issues that surround attempts to compare allelic data between multiple organisms. In order to explore the utility of paralogous genes for purposes of phenotypic *in silico* variation characterization and prioritization, we have systematically examined the genome-wide distribution of sequence variants along the lengths of paralogous proteins.

**Figure 1:**
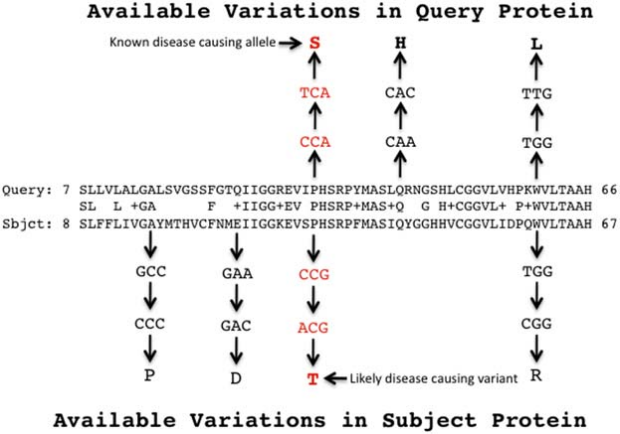
Using sequence homology to identify variant pairs. The protein encoded by a candidate disease gene (the subject in the alignment) is aligned to a paralogous protein encoded by a locus with known disease-causing alleles (the query in the above alignment). Shown in red is a paralogous variant pair. Variants in the candidate that occur in the same positions in the alignment as a known disease-causing variant in the other protein are prioritized for use in subsequent association studies.

To further test the clinical relevance of these data, we have also assembled a collection of known disease-causing variations drawn from OMIM [11] and HGMD [12]; both of these databases provide extensive documentation of disease-causing sequence variations. We then mapped each of these variations to their gene annotations and protein sequences. This dataset has allowed us to identify and characterize pairs of variations that occur at homologous positions within human disease genes (Figure 1). We find that sequence variants co-occur at aligned amino acid pairs more frequently than expected by chance, suggesting that similar functional constraints on paralogous protein sequences result in coordinated distributions of both disease and non–disease-causing variants along their lengths. Moreover, our disease-gene analyses demonstrate that if one member of a variant-pair is disease-causing, its partner is likely to be disease-causing as well. Thus, knowledge of a sequence variant's paralogous relationships is useful for purposes of *in-silico* identification of novel disease-causing alleles.

## Results

### Genome-wide analysis of dbSNP polymorphisms

In order to investigate the genome-wide distribution of coding sequence variants within paralogous genes, we used two methods to assemble sets of paralogous genes. First, we identified a set of best-hit gene pairs, e.g. every pair of genes whose proteins hit one another with a BLASTP Expect<1e^−6^. In total this set contained 17,111 human genes (termed ‘Best-hits’). Second, we identified a sub-set of 7,368 reciprocal best-hit (BLASTP Expect<1e^−6^) proteins, which is an even stronger criterion for paralogous genes; we term these Reciprocal Best-hit gene pairs. The motivation behind this procedure was to test two definitions of paralogs: a less-stringent and a more stringent. Defining paralogs simply as “Genes that have arisen by gene duplication events in an organism and are transmitted to offspring as a gene family” [10], overlooks the fact that it is possible to distinguish two types of paralogs by homology: those that are merely homologous to one another, and those that are each others best hit, e.g. reciprocal best hit pairs. These are more similar to one another than they are to any other member of their gene family. Thus the two sets of paralogs (Table 1) allowed us to ask if the positions of coding variants might be more correlated among reciprocal best-hit pairs than for best-hits; in fact both sets show very similar correlations in variant positions.

**Table 1:** ODDs scores associated with different types of variant pairs.

Dataset	Genes	% Similarity	Syn.	Non-syn.	Non-con.	Con.	Frame-shift
Reciprocal Best-hits	7,368	74.5	33.7	31.7	95.3	64.3	200.4
Best hits	17,111	69.1	32.2	31.3	89.3	62.1	218.2

Genes: number of genes in the dataset. % Similarity: average value for the dataset's aligned proteins. Syn: synonymous variants. Non-syn: non-synonymous variants (pooled variants from the other classes of variant, including nonsense variants). Non-con: non-conservative substitutions. Con: conservative substitutions. Frame-shift: frameshift inducing indels. Values in the table are ODDs scores (observed number of variant pairs/expected number of variant pairs).

Next we characterized all dbSNP reference polymorphisms [1] mapped to these 17,111 genes, first determining if they mapped to the gene's annotated protein sequence(s) in GenBank. As many of these gene-pairs encoded very similar protein sequences, we restricted our analyses to dbSNP variants with a map weight of class 2 in dbSNP—variants that can be uniquely mapped to a single location with the genome (for details see ftp://ftp.ncbi.nih.gov/snp/00readme.html). In total, this procedure gave us a set of 109,989 coding variants. We then analyzed each sequence variant's impact on the protein sequence (see Materials & Methods). We first characterized each variant according to whether it produced a synonymous (43,467), or non-synonymous (66,522) change, further breaking the non-synonymous variants into five sub-classes: conservative amino acid substitutions (30,690), non-conservative substitutions (24,070), nonsense (stop codon producing) variants (1,148), frameshift variants (9,895) and in-frame indels (719).

We then aligned each paralogous gene pair's protein sequences using BLASTP [13],[14], and used these alignments to identify aligned variants (see Figure 1 for an overview of the algorithm). In total we found 4743 aligned variants. The ODDs scores for non-conservative, conservative, and synonymous pairs were 89.3, 62.1, and 32.2 respectively. All were significant at P≪1×10^−4^ (Table 1). The ODDs score for all non-synonymous pairs, conservative and non-conservative substitutions combined, was 31.3 (P≪1×10^−4^). Surprisingly, this was lower than the individual ODDS scores for variants causing conservative and non-conservative amino acid changes. We first sought to explain this phenomenon by asking if it is due to the fact that some types of non-synonymous changes are mutually excluded from occurring at the same position in a codon, e.g. nonsense mutations cannot occur at the first and second position of a methionine codon, but amino acid substitutions can. To test this hypothesis, we regrouped the variant pairs by aligned amino acid rather than the more stringent criterion of their having the same position within the aligned codons. Interestingly this had little effect on either the ODDs scores, or their relative magnitudes to one another. Thus, it appears that the different subclasses of non-synonymous variants have distinct distributions along the aligned proteins.

We also observed a correlation between the degree of sequence similarity and fraction of aligned variant-pairs. The Spearman correlation coefficient [15] between the odds ratio (i.e. the ratio of the observed to expected numbers aligned variant-pairs) and the average number of bits per aligned position of two aligned paralogous proteins was 0.47 (P<0.001) among the 17,111 best-hit protein pairs. In other words, the more similar the to paralogous proteins, thee more correlated the positions of their sequence variants. Taken together these results suggest that similar functional constraints together with similar positions of synonymous codons in paralogous protein sequences combine to result in coordinated, class-specific distributions of variants along their lengths.

### Disease genes and their polymorphisms

Next we asked if paralogous polymorphisms can be used to identify disease-causing variants. To do so we used a set of 2,244 curated human disease-genes (the “Omicia disease gene set”), which have been documented in the literature as playing a causative role in one or more human diseases. This list of genes includes and extends a human disease gene set previously published by Jimenez-Sanchez [16] that contains 923 genes. The complete list of genes and their variants is available at http://www.yandell-lab.org/publications/variant_data.htm.

For these disease gene analyses we used a different set of sequence variants—one consisting of 35,292 coding sequence variants in 2,244 disease genes drawn largely from OMIM, HGMD and dbSNP (see Materials & Methods for details). The dataset contains:

- 4,120 OMIM variants: 4,103 non-synonymous (incl. 1,359 conservative substitutions, 1,695 non-conservative substitutions, 1,049 nonsense) and 17 synonymous variants.
- 17,467 HGMD variants: 12,312 non-synonymous (incl. 4,338 conservative substitutions, 7,811 non-conservative substitutions, and 163 nonsense variants) and 5,155 indel variants.
- 13,858 dbSNP variants: 7,268 non-synonymous (incl. 4,063 conservative substitutions, 3,058 non-conservative substitutions, 147 nonsense variants) and 6,590 synonymous variants.

Interestingly, more than 25% of the OMIM alleles are nonsense, meaning they result in a termination codon, while the HGMD and dbSNP sets contain a very small percentage (<5%) of nonsense alleles. OMIM deletion and insertion mutations were not included due to ambiguities in the entry format. Database entries that could not be mapped to the current annotated protein were also excluded from the dataset (see Materials & Methods for details).

As our disease gene set contained few genes whose proteins were reciprocal best hits, we instead examined the frequency of aligned variant pairs between each gene and its best hit (1448 gene pairs) within the set of disease genes. We first calculated the global correlation (using an odds ratio as above) in variation positions among paralogs for the 2,244 Omicia disease genes, using the 28,691 non-synonymous and 6,607 synonymous variants located within these genes. The results of this analysis are shown in Table 2. If we align polymorphisms from dbSNP against polymorphisms from dbSNP, the odds ratio for paralogous mis-sense pairs (pooled non-conservative and conservative substitution pairs; see Table 2) is 9.5 (P≪1×10^−4^). This means that, among Omicia disease genes, non-conservative and conservative polymorphisms from dbSNP co-occur as paralogous pairs 9.5 times more frequently than expected by chance. The value was 6.1 for synonymous variants. If we only consider disease-causing variants aligned to other disease-causing variants, the ratio is 8.8 (P≪1×10^−4^). The tendency of dbSNP mis-sense variants to pair with known disease-causing variants is less: the ODDs ratio is 2.2 (P≪1×10^−4^).

**Table 2:** ODDs ratios for disease-gene variant pairs.

DATABASE	MIS-SENSE	SYNONYMOUS
dbSNP vs. dbSNP	9.5	6.1
all disease vs. all disease	8.8	N/A
all disease vs. dbSNP	2.2	N/A

Column 1 lists the database of origin for each member of the variant pair. “all disease” means known disease-causing variants from OMIM and HGMD. Columns 2 and 3 give the odds ratios (observed/expected) for screening every gene from the Omicia disease gene set for paired variants using pooled non-conservative and conservative substitutions (here termed ‘MIS-SENSE’) and synonymous variants from the respective databases. P≪1e^−4^ for all values.

As was the case for best-hit paralogous protein pairs, we found that for known disease associated genes the sequence similarity of the two aligned proteins is also positively correlated with the number of their aligned variants (Spearman correlation coefficient R = 0.32 for the reciprocal best pairs, R = 0.47 for best-hits P<0.001). In summary, these results show that even though the proteins encoded by the disease gene pairs were on average less similar to one another than the reciprocal best hits used in our genome-wide investigations above (58.5% vs. 74.2% respectively), the same correlations still exist as to where sequence variants occurred along the lengths of their proteins. Moreover sequence variants known to be associated with human disease tend to align with one another more frequently than phenotypically uncharacterized polymorphisms from dbSNP.

### Disease-causing variant pairs preferentially align with one another

Next we determined whether or not disease-causing alleles tend to pair with one another more often than expected by chance. In other words, if one member of an aligned variation pair is disease-causing, is its partner likely to be disease-causing as well? In order to test this hypothesis we first chose a random set of 7000 known, disease-causing variations from among the 15,203 non-conservative and conservative (mis-sense) variants derived from HGMD and OMIM in the Omicia disease gene set; we called this Set A. We also randomly chose 7,000 of non-conservative and conservative substitution variants from among the 7,268 dbSNP non-synonymous variations located in these same genes; we called this Set B. We then assayed how often alleles in Set A and Set B were found paired with the remaining 8,203, known HGMD and OMIM disease-causing, conservative and non-conservative substitution variants not included in either Set A or Set B; these were our control set, Set C. Overall we observed an 86% enrichment (6.6-fold±0.11; P≪1×10^−4^) of OMIM and HGMD alleles from Set A paired with disease-causing OMIM and HGMD alleles in Set C, compared to dbSNP variants from Set B paired with OMIM and HGMD alleles in Set C. Thus, known disease-causing alleles tend to preferentially align with one another. This finding demonstrates that on average, choosing uncharacterized variations aligned to known disease variants will enrich 6.6-fold for clinically significant variations.

We repeated the experiment using only non-conservative variants, and once again using only conservative ones. The same trends were observed. The enrichment for Set A–C pairs was 14-fold±0.19 (P<0.0001) for conservative variants, and even greater for non-conservative variants (19-fold±0.19; P<0.0001). Thus pooling non-conservative and conservative polymorphisms lowers the relative enrichment for disease-causing pairs, a result consistent with our earlier genome-wide paralog analyses. In summary, these results demonstrate that disease-causing variants tend to associate with one another to the exclusion of non-disease-causing variants—implying that novel variants in gene A aligned with known disease-causing variants in gene B (Figure 1) are on average more likely to be disease-causing than are novel polymorphisms occurring elsewhere in gene A.

## Discussion

We have performed the first analysis of the distribution of DNA sequence variants within the protein coding portions of paralogous genes. Our data show that (1) protein sequence variants, both synonymous and non-synonymous, tend to occur with high frequency at homologous positions within paralogous proteins; (2) that different subclasses of variants have distinct distributions along the aligned proteins; and (3) that disease-causing variants also tend to pair with one another. Overall, the magnitude of the correlation in variant positions is correlated with the sequence similarity of the two proteins. These facts suggest that similar patterns of codon usage and functional constraints combine to produce correlations in the locations of variants along the lengths of paralogous proteins. This coordination includes not only common (MAF>2%), synonymous variants, without phenotypic consequences, but also extends to rare, disease-causing alleles.

We also discovered that different subclasses of variant have distinct distributions along the lengths of paralogous proteins. Two facts support this conclusion. First, we observed differing tendencies of variant sub-classes to pair with one another (Table 1). Among best-hit paralogous proteins, the ODDs scores for synonymous, non-synonymous, conservative, and non-conservative variants are 32.2, 31.3, 62.1 and 89.3 respectively. Second, combining classes always depresses the ODDs score. Thus it appears that each subclass of variant occurs in a specific pattern along the lengths of paralogous proteins, with non-conservative variants having the most highly correlated distribution. One possible explanation of the different distributions is that purifying selection acts to restrict non-synonymous substitutions to a subset of positions in the two proteins, while synonymous variants are free to occur at a greater number of positions; hence the lower ODDS score for synonymous pairs.

Disease-causing variants also tend to align with one another. Moreover, they do so to the exclusion of phenotypically uncharacterized variants in dbSNP. Overall, disease-causing variants are 6.6-fold (P<1×10^−4^) more likely to pair with one another than with non-synonymous dbSNP variants. When disease-causing variants producing conservative and non-conservative amino acid changes are considered separately, the enrichment is even more pronounced: 14- and 19-fold, respectively. As the dbSNP database presumably contains some undiscovered disease-causing variants, these odds ratios are likely lower bounds; thus the trend is quite robust. Though speculative, one possible explanation of these facts is that similar functional constraints in paralogous proteins restrict rare, disease-causing variants to a few homologous positions where particular amino acid substitutions produce the disease phenotype. In any case, like other classes of variant, rare, disease-causing variants in paralogous proteins also tend to pair.

**Figure 2:**
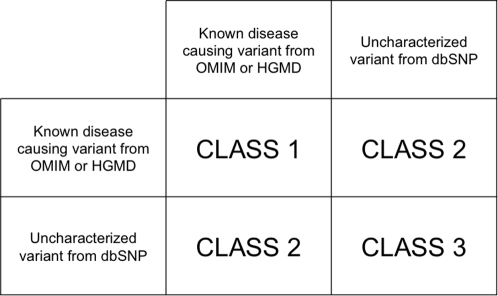
Classification system for variant pairs.

Alignments of paralogous proteins and their variations provide a novel resource for functional genomics. Consider that aligned variation pairs can be divided into three basic classes depending on whether their members are known to be disease-causing or not; Figure 2 provides a summary of this classification system. Each class 1 pair, for example, relates a pair of known diseases, both caused by mutations at equivalent positions in two paralogous proteins. Table 3 shows a sample set of class 1 pairs. Similar changes in similar proteins suggest similar biochemical etiologies, and in some cases overlapping disease phenotypes. Patients with Menkes and Wilson disease, for example, both suffer from abnormalities in copper metabolism [17]–[19]. Likewise Alagille and Marfan syndrome Type I are both associated with spinal, vision, and circulatory abnormalities [20]–[25]. These facts demonstrate how variant pairs can be used as starting points in the search for latent knowledge in disease literature and databases. In other words, therapies and drugs used to treat disease symptoms caused by mutations in one member of a pair might prove efficacious in treating the other disease as well. No doubt, a myriad of issues including time and place of gene expression will complicate such simple conclusions. Nonetheless, these data show how paralogous disease genes and their variant-pairs can be used for hypothesis generation and as points of departure for further clinical research. Similarly, extending this procedure to include paralogs of known disease genes, that are not themselves yet associated with any disease could be used to identify new disease gene candidates, and to identify uncharacterized variants within them likely to have phenotypic consequences.

**Table 3:** Selected Class 1 SNP pairs.

Gene A	ID of SNP in Gene A	Disease assoc. with Gene A	Gene B	ID of SNP in Gene B	Disease assoc. with Gene B
FGFR2	HGMD:CX972741	Pfeiffer syndrome	FGFR3	HGMD:CM950470	Thanatophoric dysplasia
JAG1	HGMD:CD993777	Alagille syndrome	FBN1	HGMD:CM972811	Marfan syndrome
ATP7A	HGMD:CM940140	Menkes syndrome	ATP7B	HGMD:CM970138	Wilson disease
ABCA1	HGMD:CM993803	Tangier disease	ABCA4	HGMD:CM990025	Stargardt disease
CFTR	HGMD:CM940275	Cystic fibrosis	ABCC8	HGMD:CM981883	Hyperinsulinism

Columns 1 & 4 give the gene symbols for two paralogous disease-causing genes. Columns 2 & 5 give the IDs of the two variants that comprise the Class 1 pair. Columns 3 & 6 list the diseases most commonly associated with the two paralogous variants.

Another use for variant-pairs is in variation prioritization for association studies and clinical validation studies. Current AAS methodologies [4] such as SIFT [5] and PolyPhen [8] look for those sequence changes likely to disrupt conserved regions. Obviously, many disease alleles do result in violent changes to conserved portions of proteins—frameshift, and nonsense mutations for example. Nevertheless, some alleles are more subtle: For some proteins, conservative changes in poorly conserved regions may be the only tolerated changes; in other cases, even non-conservative changes that destroy protein function may not be disease-causing, especially for non-essential, redundant genes. These considerations suggest that a hybrid approach to variant prioritization might prove effective, one that used conservation together with information about the locations of uncharacterized variants relative to other disease-causing alleles, e.g. Class 2 variants in Figure 2. Our analyses suggest that such an approach might improve the performance of AAS approaches, especially for genes with a closely related paralog known to be a human disease gene.

Our results naturally raise questions as to the origins of variant pairs. One possibility is “common descent”, but this seems unlikely. Widely accepted models for gene duplication [26]–[28] generally assume that paralogous genes arise from one or a few ancestral duplication events, which are later fixed in the population as a result of positive selection. No matter how numerous the variations at each position in the progenitor protein, a duplication event will fix a single variant at each position in the new paralog. Thus, it seems more likely that variants in the duplicated gene will have arisen after the duplication event, especially for more ancient duplications—and that the correspondence in their locations is a product of similar selective pressures acting on both paralogs.

Another potential explanation for the trends we have observed is gene conversion [26],[29],[30], which may provide another source of coordinated variation among highly conserved paralogous genes. Repeated recombination between the new duplicate and its ancestor within a population might furnish the newer gene with many of the same variants as its parent. Novel variants in the duplicate might also be passed to its ancestor as well. In principle, disequilibrium and/or similar variant frequency spectra at aligned amino acids might be used to distinguish this model from the one proposed above, with disequilibrium and similar variant frequency spectra construed as supporting the gene conversion model. However, such analyses lie outside the scope of the present paper. Whatever the ultimate cause of the phenomenon, our results clearly demonstrate that variations tend to occur at equivalent positions in paralogous proteins. This fact provides new avenues for DNA variant prioritization and for clinical studies.

## Materials and Methods

### Datasets

The DNA variants used in this study were obtained as follows. For the “Genome-wide analyses of dbSNP polymorphisms”, variant data was retrieved from NCBI's dbSNP FTP site and stored in a MySQL database as described below. All fields from the chromosome reports (ftp://ftp.ncbi.nih.gov/snp/organisms/human_9606/chr_rpts) for chromosomes 1–22, X and Y were loaded into a database table. Fasta files of the sequence variants were downloaded from ftp://ftp.ncbi.nih.gov/snp/organisms/human_9606/rs_fasta. Each variant's ID and allele were parsed from the fasta header and loaded to a table in the database. Flatfile dumps of the ANS1 formatted dbSNP data were retrieved (ftp://ftp.ncbi.nih.gov/snp/organisms/human_9606/ASN1_flat) and parsed for contig accession and version, variant coordinates, location type and orientation. These fields were also loaded into a database table. Finally, a computational biology software library for genome annotations called CGL [31] (www.yandell-lab.org/cgl) was used to extract contig based gene coordinates for each human gene from NCBI's release 36.2 of the human genome annotation (ftp://ftp.ncbi.nih.gov/genomes/H_sapiens) and this data was loaded into the database. Structured Query Language (SQL) was used to query the database for every variant that was contained within the boundary of each gene's coordinates and a report was generated for each gene's variants containing their contig ID, gene symbol, RefSNP ID [1], location of variant on the contig, map weight of the variant, allele, location type, and orientation.

The variants used in the disease gene analyses were obtained from 3 sources: OMIM [11], HGMD [12] and dbSNP [1]. In each case we used a set of 2244 human disease genes based upon a list published in Jimenez-Sanchez et al [16]. This list of 923 genes was extended to include every gene from OMIM or HGMD with at least one sequence variant having a peer-reviewed publication showing its involvement in or association with a human disease. OMIM alleles in these genes were selected as follows. Disease-causing alleles and sequence variants implicated in disease predisposition were parsed from OMIM XML documents. Unfortunately, the positions of these variants on the currently annotated protein sequence are often unknown, as OMIM indexes its coding variants according to the amino acid they alter on the protein sequence reported in the publication, rather than the currently annotated protein sequence. The currently annotated protein often differs from these sequences. In order to circumvent this issue, we developed a mapping process to move the OMIM alleles forward to the current annotations. It works as follows. OMIM alleles are documented by the change they cause, e.g. H35K would refer to a variant that changes a histidine located at position 35 to a lysine. Usually we were able to obtain this information for several variants at various positions along the protein. We then asked if there was a single offset that would map each variant to the currently annotated protein. Consider two variants annotated as H35K and W87S. Although the currently annotated protein might not contain either of these amino acids at the these positions, in many cases adding or subtracting a constant value from both of the OMIM locations will be bring them into register with the currently annotated protein sequence. Assuming *n* alleles are available, the probability that this would occur by chance would be around (1/20)*^n^*, neglecting amino acid frequency biases. To control for this factor we automatically identified low complexity proteins and manually reviewed each placement on these proteins. In total we were able to map more than 80% of OMIM alleles by this procedure. HGMD [12] allele information was obtained from HGMD as XML documents. These were post-processed, and checked for agreement with the current GenBank annotations. Variants from dbSNP were chosen on the basis of their frequencies (MAF>2%). A publication on the details of this algorithm is in preparation.

### Classification of variants

Once our variant selection process was complete, we then classified each coding variant, using a CGL-based script [31], into one of 6 categories on the basis of the change to the protein: synonymous, conservative substitution, non-conservative substitution, nonsense, frameshift, and in-frame insertion or deletion. Designation as conservative versus non-conservative was based upon the BLOSUM 62 matrix [32]; changes with a score≥0 were considered conservative, those less than 0, non-conservative.

### BLAST searches

All blast searches were carried out using WU-BLASTP (http://blast.wustl.edu) with the following command line: blastp db query –B = 1000 –E = 0.0001 –Z = 300000. Hits were parsed at E<1e^−6^.

### Conversion of GenBank annotations

GenBank annotations (version 36.2) were downloaded from ftp.ncbi.nlm.nih.gov/genomes. These were converted from GenBank format to BioChaos XML documents using the Bio-Chaos software library (http://www.fruitfly.org/chaos-xml). The resulting XML documents were used as inputs, together with the variant data described above, to a CGL-based pipeline. Variant locations were first classified as coding or non-coding, and then further classified according to the type of change to the annotated protein sequence. Variants were related to one another using BLASTP protein alignments and CGL was used to map the variants onto the protein alignments, and onward to their implied aligned codons. This allowed us to keep track of both the amino acid and the corresponding underlying nucleotides. Thus we were able to ascertain when variants mapped to same amino acid and when they mapped to the equivalent position within the codon as well.

### ODD scores and significance calculations

We followed an established procedure to calculate odds scores for aligned amino acids [32] and simply adapted it to aligned variants. The expected frequency of variant-pairs was obtained by tallying the number of variants contained in the aligned portions of each query protein, and dividing that value by the total length of the BLASTP alignments. The same calculation was also carried out for each subject protein sequence. The product of these two frequencies gives the expected frequency of variant pairs. Next, the number of aligned variant pairs (the observed) were tallied and then divided by the total length of the BLASTP alignments, to give the observed frequency of aligned variants. The reported ODDs scores are the ratios of the observed and expected frequencies. This simple model for the expectation provides a means to measure the tendency for variants to pair, and to quantify the magnitude of the trend in order to estimate its utility for prioritization and data mining purposes. Although it might be possible to formulate an expectation model that takes into account the relative contributions of the genetic code and purifying selection, this would not provide a means to measure the tendency of variants in paralogous proteins to pair—our goal. To see why, consider that under an expectation model that correctly accounted for the relative contributions of codon-substitution patterns and purifying selection, we would expect an odds score of 1.0, i.e. the observed frequency would equal the expectation.

The Statistical significance of the ODDs scores was estimated by simulation. The frequencies of variant pairs used in the expectation calculation were used to produce two strings of 0 s and 1 s equal to one-tenth the length of total length of the BLASTP alignments, wherein a 1 represented the occurrence of a variant. Perl's rand function and the frequencies of variant pairs in the BLASTP alignments were used to produce these strings. 1 s appearing in both strings at the same offset were scored as aligned. This simulation was repeated 10,000 times; if none of the simulations had an ODDs score equal to or greater than the reported value, then the reported value was considered significant at P<1×10^−4^. In practice the simulated ODDs scores never climbed above 1; a rather obvious fact, as given the length of the strings involved (L∼1×10^6^), the variance from the expected value is very small. Because we used a length 1/10 the actual in our simulations (in order to speed the calculation), the actual P value is likely much less than 1×10^−4^. This approach was also adapted to estimate the statistical significance of the enrichments seen in our disease-gene analyses. Spearman correlation coefficients [15] were calculated using the Statistics-Rank-Correlation module from CPAN.org (www.cpan.org). Their Statistical significance was calculated by randomizing the data some number of times (usually 1000) and then asking if any correlation of the same magnitude ever appeared by chance.
